# Polysaccharide-Based Hydrogels for Wound Dressing: Design Considerations and Clinical Applications

**DOI:** 10.3389/fbioe.2022.845735

**Published:** 2022-03-07

**Authors:** Rongwei Cui, Luhan Zhang, Rongying Ou, Yunsheng Xu, Lizhou Xu, Xiao-Yong Zhan, Danyang Li

**Affiliations:** ^1^ Research Center, The Seventh Affiliated Hospital, Sun Yat-sen University, Shenzhen, China; ^2^ Department of Dermatovenereology, The Seventh Affiliated Hospital, Sun Yat-sen University, Shenzhen, China; ^3^ Department of Gynaecology and Obstetrics, The First Affiliated Hospital of Wenzhou Medical University, Wenzhou, China; ^4^ ZJU-Hangzhou Global Scientific and Technological Innovation Center, Hangzhou, China; ^5^ Department of Materials, Imperial College London, London, United Kingdom

**Keywords:** polysaccharide hydrogel, wound dressing, design considerations, clinical applications, physicochemical properties, biological functionalities

## Abstract

Wound management remains a worldwide challenge. It is undeniable that patients with problems such as difficulties in wound healing, metabolic disorder of the wound microenvironment and even severely infected wounds *etc.* always suffer great pain that affected their quality of lives. The selection of appropriate wound dressings is vital for the healing process. With the advances of technology, hydrogels dressings have been showing great potentials for the treatment of both acute wounds (e.g., burn injuries, hemorrhage, rupturing of internal organs/aorta) and chronic wounds such as diabetic foot and pressure ulcer. Particularly, in the past decade, polysaccharide-based hydrogels which are made up with abundant and reproducible natural materials that are biocompatible and biodegradable present unique features and huge flexibilities for modifications as wound dressings and are widely applicable in clinical practices. They share not only common characteristics of hydrogels such as excellent tissue adhesion, swelling, water absorption, *etc.*, but also other properties (*e.g.,* anti-inflammatory, bactericidal and immune regulation), to accelerate wound re-epithelialization, mimic skin structure and induce skin regeneration. Herein, in this review, we highlighted the importance of tailoring the physicochemical performance and biological functions of polysaccharide-based hydrogel wound dressings. We also summarized and discussed their clinical states of, aiming to provide valuable hints and references for the future development of more intelligent and multifunctional wound dressings of polysaccharide hydrogels.

## Introduction

Wound is the damage of tissues and/or organs accompanied by the destruction of the integrity of the skin or mucous membrane (Gupta et al., 2019), which plays an extremely important role in preventing water loss and blocking the invasion of harmful substances and pathogenic microorganisms as a significant interface between the body and its surroundings (Zhou et al., 2021). Once a wound is developed, intervention is necessary because the healing process is highly coordinated, including long-term anti-infection, various forms of cell rejuvenation such as collagenation, epithelialization, and tissue remodeling (Brumberg, Astrelina, Malivanova, & Samoilov, 2021).

Wound dressing is an important tool for intervention in wound healing which has been an important aspect of biomedical materials research. In the past, most wounds were dealt with conventional wound dressings which composed of fabric materials. Although to a certain extent they can protect the wound from contamination and absorb the wound exudate, they were not able to provide an appropriate environment for tissue regeneration. In addition, the frequent changes of the dressing to prevent the maceration of healthy tissue may cause more damage and pain (Gupta et al., 2019). Therefore, advanced dressings have been exploited such as hydrogels, thin films (membranes), nanofibers, foams, and sponges to overcome the shortcomings of conventional dressings (Y. Chen et al., 2020) (Fan et al., 2021), providing not only a physical barrier against secondary infection, but also a compatible physiological environment. Among all these advanced dressings, hydrogel becomes a rising star in rapid wound healing (Jacob et al., 2021), by providing a biocompatible, moisture, and antibacterial interface between the dressing and the wound (Zhang et al., 2019).

Polysaccharide-based hydrogels composed of natural polymers, such as alginate, cellulose, chitosan, hyaluronic acid, *etc.* were identified to better meet the requirements of wound healing compared with other hydrogels (Aduba & Yang, 2017; Srivastava et al., 2021). Firstly, the backbone of polysaccharide polymer contains a large number of hydroxyl and carboxyl groups (Beaumont et al., 2021), which enables the formed hydrogel higher water content and better swelling performance, resulting in unparalleled moisturizing properties to absorb tissue exudate and negligible adhesion to the wound tissue (Ahmad et al., 2019). Moreover, the well-established modifications of the polysaccharide polymers *via* Schiff base Reaction (Mo et al., 2021), Dynamic B−O Bonds (Aeridou et al., 2020), Diels–Alder reaction (Trombino et al., 2019) and so forth with carboxyl and hydroxyl groups allow tailoring the mechanical properties of hydrogels to adapt to the different elasticity requirements of different tissues. Additionally, the porous structures and multifunctionalities of polysaccharide polymer were favorable in encapsulating various proteins, peptides, nucleic acids, and lipid drugs, *etc.* for antibacterial, anti-inflammatory and regenerative purposes in wound healing (Jacob et al., 2021) (Kuznetsova et al., 2020). Besides, polysaccharides are components of the extracellular matrix (Rosellini et al., 2018), which render them excellent biocompatibility and mimicking the functions of the extracellular matrix to facilitate the wound healing process. Finally, polysaccharide polymers are abundantly available and cheap, which makes them very suitable for large-scale production and commercialization (Camponeschi et al., 2015; Graham et al., 2019). Thus, polysaccharide-based polymers have great potentials and commercial values in the application of wound healing (Witzler et al., 2019).

In this review, we discussed and summarized some of the important physicochemical properties, including mechanical, rheological, molding time, swelling and moisturization of the obtained polysaccharide-based hydrogels via their preparations as wound dressings. The biologically active roles that the polysaccharide hydrogels played were also highlighted. In addition, we reviewed and analyzed the current states of polysaccharide-based hydrogel wound dressings that have been approved by FDA or in clinical trials. We hope this short review can provide some valuable hints and references for the future development of more intelligent and multifunctional polysaccharide hydrogel wound dressings.

## Design Considerations

Wound healing is a complicated process including four overlapping but distinct stages: hemostasis, inflammation, proliferation, and remodelling. Polysaccharide-based hydrogels can be extensively involved in these four processes (Gurtner et al., 2008). Hemostasis occurs immediately after tissue damage which components of platelet aggregation and fibrin clot formation. Polysaccharide-based hydrogels often act as accelerators at this stage because the positive charge of polysaccharide polymers such as chitosan can associate with the negatively charged cell surface via electrostatistic interaction, which quickly causes the aggregation of red blood cells and stop the bleeding (Liang et al., 2021). Inflammation occurs at the same time that the inflammatory response caused by neutrophils and macrophages can remove foreign bodies, bacteria, and damaged endogenous tissues as well as secrete chemokines and growth factors to further attract cells. Polysaccharides have been confirmed to act as immunomodulatory substances to regulate the appropriate inflammatory response at the wound site (Tang et al., 2022). Proliferation begins 2–10 days after injury. There are complex changes in the wound site during this period such as the migration of keratinocytes, the formation of the new blood vessels, the replacement of fibrin matrix by granulation tissue and the emergence of new substrate. A large number of studies have revealed that the polysaccharide hydrogel dressing achieves a better healing effect by promoting re-epithelialization (Liang et al., 2021). Remodelling begins since the completion of re-epithelialization, during this stage, all of the processes activated after injury wind down and cease (Gurtner et al., 2008).

The role of polysaccharide hydrogel for managing healing is also complex, in which the physicochemical properties (e.g., mechanical, rheological, swelling, moisturizing and heat absorption properties, *etc*.) of the dressing hydrogels are account for both their performance and applicability for clinical applications and commercialization.

### Mechanical and Rheological Properties

**FIGURE 1:**
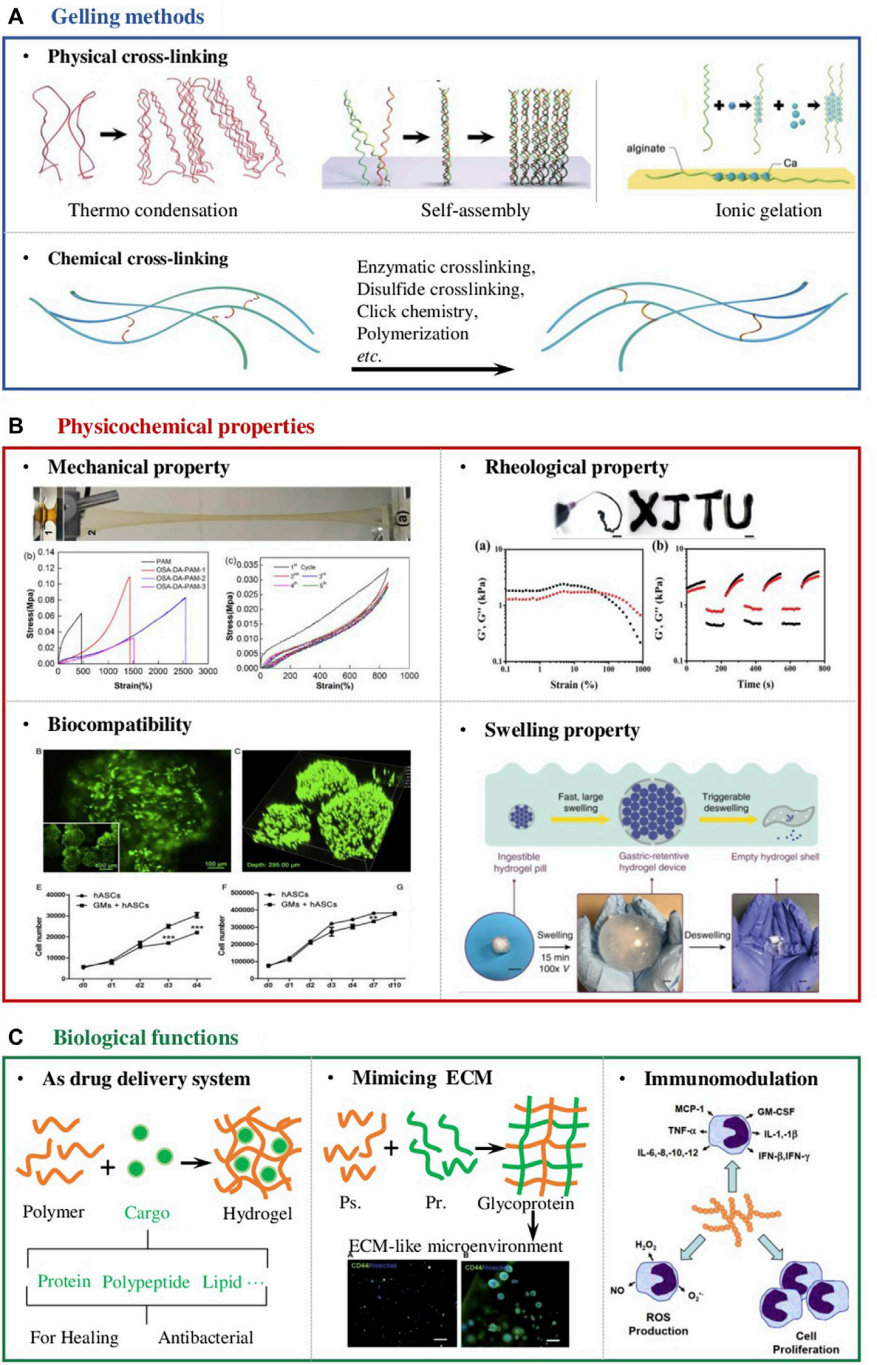
Design considerations of polysaccharide-based hydrogel wound dressings. **(A)** Schematic diagram of the two main cross-linking methods of polysaccharide-based hydrogels, namely physical and chemical cross-linking (Zhang and Khademhosseini, 2017) **(B)** The physicochemical properties that should be considered in the design of hydrogel wound dressings include but are not limited to mechanical properties (Chen et al., 2018), rheological properties (Zhao et al., 2020), biocompatibility (Zeng et al., 2015) and swelling properties (Zhu et al., 2019). **(C)** The unique biological properties of the polysaccharide-based hydrogel enable it to manage wounds; Firstly, they can serve as a drug delivery system to deliver antibacterial agents, growth factors, stem cells and so on. Secondly, polysaccharide molecules are the main components of the extracellular matrix (ECM) (Bystroňová et al., 2018). When designing wound dressing, they can be compounded with proteins to form glycoproteins to provide cells in the wound with a microenvironment similar to the ECM. In addition, polysaccharide molecules themselves can participate in the immune modulation process in the body, so designing a hydrogel with an appropriate degradation rate can promote wound healing from the perspective of immune regulation (Schepetkin & Quinn, 2006; Shah et al., 2021).

In chemistry, the gelation process is known as the progressive crosslinking of polymer chains in the reaction system into one gigantic molecule with ‘infinite’ size(Gao, Peng, & Mitragotri, 2021). The abundance of functional groups of polysaccharides enables the formation of hydrogels by physical (Iijima, Hatakeyama, & Hatakeyama, 2021) or chemical crosslinking (Figure 1A). (Ahmad et al., 2019; Nie et al., 2019) Physical method generally refers to the cross-link of polysaccharide polymers by weak interaction forces such as H-bonding, Van der Waals interactions, hydrophobic forces and molecular entanglements (Francesko et al., 2018; Iijima et al., 2021), whereas chemical gelling often involves the covalent bonding between polysaccharide polymers or polysaccharide polymer and the corresponding crosslinker, including condensation reactions, enzymatic crosslinking, disulfide crosslinking, click chemistry, polymerization, *etc* (Aeridou et al., 2020; Meng & Edgar, 2016; Mo et al., 2021). Whether it is chemical crosslinking or physical crosslinking, the chemical mechanism of its crosslinking is forming interchain linkages, native or pre-anchored functional groups on one polymer chain must react with the corresponding groups on another chain using stepwise crosslinking chemistries (Gao et al., 2021). In the following content, we focused on discussing the impact of gelling methods on the physicochemical properties of polysaccharide-based hydrogel for wound dressing (Figure 1B). The requirements of the mechanical and rheological properties of the dressing materials are determined by where they apply (Figure 1). Certain adhesion properties and tensile strength are essential for wounds undergo unavoidable movements (Yang et al., 2021). For instance, wound dressings used on joints should be tough and fatigue resistant, which are often synthesized by chemical crosslink (Popa et al., 2015). Zifeng Yang *et al.* reported a cellulose-based hydrogel prepared by chemically crosslink with cationic polyelectrolytes. The obtained hydrogels possessed high tensile strength (21–51 kPa), large tensile strain (899–1,047%), and good compressive property, ensuring the hydrogel adapted to frequent joint movements without falling off (Yang et al., 2021). Injectability or thixotropy of the dressing hydrogels are generally required for wounds on organs in which the wounds can be repaired by minimally invasive means (Deng et al., 2021; Zawani & Fauzi, 2021). In some other situations, degradation of the hydrogels is required simultaneously with healing. These mentioned scenarios often require physically cross-linked hydrogels. It was reported that a chondroitin sulphate and sodium alginate complex injectable hydrogel gelled by solvent casting method. The hydrogel was subcutaneously injected for 24 h followed by histological examination. The *in vivo* wound healing results showed the upregulation of fibroblasts-like cells, collagen deposition, and differentiated keratinocytes stimulating dermo-epidermal junction (Shah et al., 2021).

### Molding Time

Physical crosslinking of hydrogel is fast, *e.g.,* ionic gelation can be done instantly. Other physical cross-linking methods such as hydrogen bonds, host-guest chemistry, hydrophobic interaction, π-π stacking interaction and coordination bonds can also be completed within hours (Ding & Wang, 2017; Kuznetsova et al., 2020). In contrast, chemical crosslinking usually take longer (Zhu et al., 2019). For irregularly shaped wounds that require the dressing materials to be glued *in situ*, physical cross-linking should be given priority in this circumstance (Ding & Wang, 2017). Juho Lee *et al.* developed an *in situ* alginate-based hydrogel-forming/NO-releasing powder dressing, which absorbed the fluids in the wound bed and gelled immediately (Lee et al., 2019). The characteristics of rapid *in-situ* gelation make the hydrogel wound dressing more portable and faster to apply.

### Swelling and Moisturization

The swelling performance and moisturization of a hydrogel are closely related to the degree of cross-linking. In general, the higher the degree of cross-linking, the poorer the swelling performance (Lou et al., 2011). Hence, for wounds that require the dressing hydrogel with excellent swelling properties to help absorb tissue exudate, it is more beneficial to apply hydrogels with a low degree of crosslinking which is often achieved by physical method. Ching-Wen Lou et al. prepared a hydrogel by cross-linking the low-methoxyl pectin with calcium ions, which was capable of absorbing tissue fluid, keeping the wound moisture. The results also showed the ratio of the formulation composition influences the water retention and swelling properties (Lou et al., 2011). In addition, other researchers have attempted to obtain double-networked hydrogels for more demanding wound healing by combining physical and chemical cross-linking of the hydrogels (T. Chen et al., 2018; Suflet et al., 2021). For instance, a sodium alginate-based hydrogel was developed with efficient self-healing ability (80% mechanical recovery in 6 h), high tensile strength (0.109 MPa), and ultra stretchability, which are considered as desirable properties and superior to previously reported tough and self-healing hydrogels for wound dressing applications (T. Chen et al., 2018). Suflet *et al.* synthesized stable chitosan-based hydrogels by combining covalent and physical cross-linking methods, which showed a relative high swelling rate and low elastic modulus (3–30 kPa). As a result, the hydrogels are soft and flexible, which are ideal candidates for oral dressings (Suflet et al., 2021).

### Biological Functions

Despite the adaptable physicochemical properties of polysaccharide-based hydrogels, the biological functions they provided to promote the process of wound healing are also crucial (Brumberg et al., 2021). Hence, design considerations of excellent wound dressings to maximize the therapeutic effects of the hydrogels should also be included (Figure 1C).

Indeed, the large number of functional groups such as carboxyl and hydroxyl groups on the backbones of polysaccharide polymers allow flexible modifications of the hydrogel to complex with different drugs and later release them *via* various cues (Cassimjee et al., 2020). It was reported that a polysaccharide-based wound dressing hydrogel was encapsulated with antimicrobial substance and wound repair substance such as anti-inflammatory drugs (Baranov et al., 2021), proteolytic enzymes (Auriemma et al., 2020), and growth factors (Delair, 2012). Deng *et al.* also reported a sodium alginate-based hydrogel embedded with semiconductor-like metal–organic frameworks encapsulating noble metal nanoparticles. The composited hydrogels showed not only remarkable bactericidal activity against both E. coli and S. aureus by the ROS generation, but also significantly accelerated wound healing (Deng et al., 2021).

Natural bio-polysaccharide macromolecules can be complexed with proteins to form glycoproteins that mimic the extracellular matrix to provide critical cues to control cellular functions as well as guide tissue repair and regeneration (Bystroňová et al., 2018; Afewerki et al., 2019). A biomimetic wound dressing that mimics the extracellular matrix, consisting of a hydrogel matrix composed of alginate and gelation was prepared. The biologically active hydrogel was shown to significantly enhance the growth/viability and attachment/spreading of human epidermal keratinocytes compared to the control. In the *in vitro* skin irritation test system, this hydrogel also presented minimal adverse effects on the reconstructed human epidermis samples (Afewerki et al., 2019).

The polysaccharide molecules released from the degradation of the hydrogels are reported to be involved in many immune processes, allowing for pro-inflammation, anti-inflammation and repair of the wound site through immunomodulation (Schepetkin & Quinn, 2006). Zeng *et al.* proved that in a macrophages/fibroblasts co-culture system, the proliferation and migration of fibroblasts were promoted in the presence of an alginate-gelatin- hydrogel complex. Moreover, the expressions of inflammatory cytokines and chemokines were improved when compared with the corresponding fibroblasts or macrophages monocultures (Zhu et al., 2019).

## Clinical Application

**FIGURE 2:**
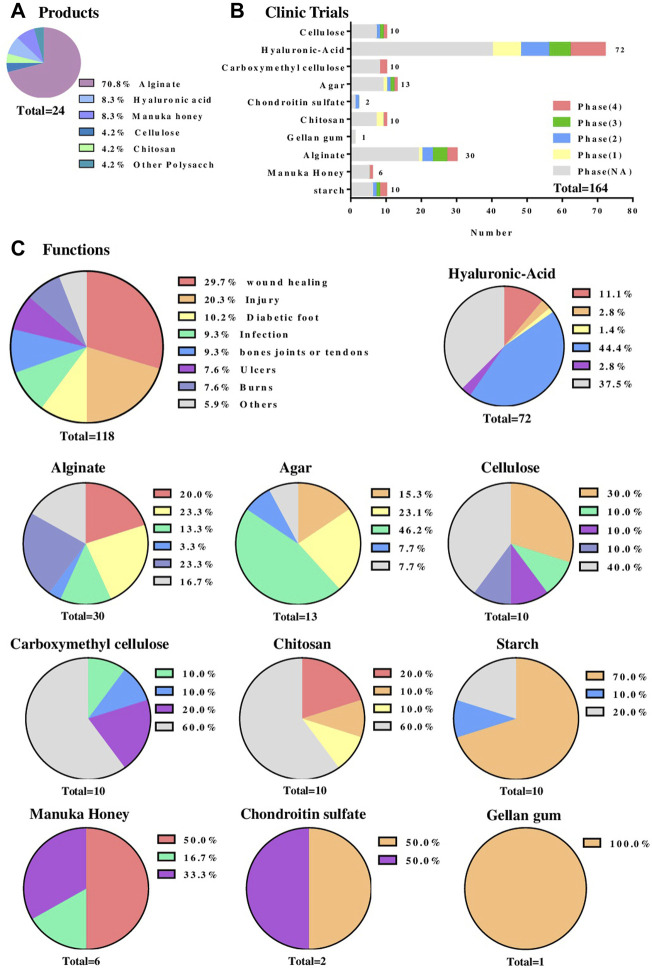
Clinical application of the polysaccharide-based hydrogel wound dressing. **(A)** A statistical chart of the main polysaccharide components of polysaccharide-based wound dressings registered with the FDA. Data Sources from *www.accessdata.fda.gov/* *.* **(B)** The statistical chart of the main polysaccharide components of polysaccharide-based hydrogel wound dressings that are undergoing clinical trials and the phase states of these hydrogels. Data Sources from https://clinicaltrials.gov/. **(C)** The types of wounds that different polysaccharide-based hydrogel wound dressings deal with which are undergoing clinical trials. The first figure is the overall function statistics. Data Sources from https://trialsearch.who.int/.

Nowadays, advanced wound care products make up around $7.1 billion of the global market and their production is growing at an annual rate of 8.3% with the market projected to be worth $12.5 billion by 2022 (Gupta et al., 2019). In the market of hydrogel wound dressings, there are 24 types of polysaccharide-based hydrogel wound dressing products registered with the FDA (Figure 2A). Including alginate, cellulose, chitosan, hyaluronic acid, manuka honey, *etc*. Among these products, alginate-based hydrogel wound dressings accounted for around 70% (17 types), which provide a moist wound environment and facilitate autolysis for partial and full-thickness wounds with large amounts of drainage and so forth. These hydrogels generally adopt the physical cross-linking method of calcium ion cross-linking, and give priority to ensuring that the hydrogels play a protective effect, and are mainly adapted to wounds that are not serious and have a normal healing process (Francesko et al., 2018; Saghati et al., 2021). In addition, there are many products designed to fulfill the drug delivery function of polysaccharide hydrogels. Those products (*e.g.,* Algicell™ Algicell^®^, Integra LifeSciences Corp. Algidex Ag®Gel, DeRoyal) are loaded with Ag^+^ to render them antibacterial effect. Metal ions such as Ag^+^ can be combined with the long molecular chain of sodium alginate through strong or weak chelation to achieve sustained release at the wound site.

The statistics from https://clinicaltrials.gov/show that till December 2021, there are 164 cases in clinical trials (Figure 2B) involving polysaccharide-based wound dressings, of which hyaluronic acid (HA) accounts for 72 cases with 10 are already in phase 4. Besides the previously mentioned merits of polysaccharide hydrogels, HA presented favorable characteristics in promoting the migration and proliferation of epithelial and endothelial cells, decreasing the inflammatory processes and enhancing the angiogenesis (Trombino et al., 2019). It was revealed in Figure 2C that polysaccharide based wound dressings are widely applicable in clinical trials for wound healing, injury, diabetic foot, infection, burns, ulcers and so on. In detail, hyaluronic acid can be used as a dressing for various wounds such as oral cavity, corneal epithelial wound, radiation dermatitis and 2-degree burn. Besides, a large number of chemical cross-linking agents such as 1,4-butanediol diglycidyl ether are used to cross-link hyaluronic acid to obtain very considerable mechanical properties (Trombino et al., 2019). Hence nearly 50% of clinical trials apply chemically cross-linked hyaluronic acid to the treatment of bones, joints and tendons wounds (Figure 2C). Meanwhile, alginate ranked second with 30 (around 18%) cases used in clinical trials (Figure 2B) and they were mainly being used for wounds caused by burns, injuries, trauma, ulcers, infections and diabetic foot, *etc* (Figure 2C). Such wounds often require physically cross-linked hydrogels because they are milder and safer, and do not have the toxic side effects of chemical cross-linking agents. This makes sodium alginate, which is highly sensitive to ionic crosslinking, stand out. Other types of polysaccharide hydrogel dressings such as agar, cellulose, chitosan, and starch were used less in clinical trials, however more often than manuka honey, chondroitin sulfate and gellan gum that used relatively rare.

## Conclusion and Perspectives

The distinctive characteristics of polysaccharide-based hydrogels such as high-water retention capacity, biocompatibility, biodegradability and tunable functionality make them competitive candidates in the application of wound dressing (Y. Chen et al., 2020). In adaptable to various types of wounds and circumstances, the design of physicochemical and biological properties of polysaccharide hydrogel dressings remains the key question to answer. Particularly, in this review we discussed the influences of different gelling methods, *i.e.,* chemical, or physical crosslinking on the mechanical, rheological, modeling time and swelling properties of the dressings that give the fundamental indications for designing appropriate wound healing materials. The porous structure and multiple functionalities of polysaccharide hydrogels enable the encapsulation of bioactive molecules or act as immunomodulators themselves, which further promoted the healing process of wounds.

In addition, we summarized the polysaccharide-based hydrogel products that are available on the market and in clinical trials in terms of the dressing materials and their clinical applications. Hyaluronic acid and sodium alginate are the most extensively used materials and will increasingly occupy the market share in the future. Nevertheless, the healing of wounds is a dynamic and complicated process that it is difficult to have a dressing that is always ideal (Brumberg et al., 2021; Zhou et al., 2021). Further studies looking into the modifications of polysaccharide-based hydrogels or the development of other types of polysaccharide-based dressings are still in need to provide more precise control of the physicochemical properties of the gels, cover a wider range of scenarios and have a more pronounced biomodulatory effect on wounds. In comparing with traditional hydrogel wound dressings, there are increasing attempts to integrate electronic components to hydrogels, in order to capture subtle changes in the wound site, allowing for more accurate and comprehensive monitoring of the healing process (Brumberg et al., 2021) (Francesko et al., 2018). There are also emerging research to fabricate microneedle-like hydrogels or render them a more varied topological surface profile to better fit the needs of diverse form wounds (Zhou et al., 2021). Ultimately, in corresponding to the idea of precision and personalization medicine, we believe the development of polysaccharide-based hydrogel wound dressings will evolve towards smarter and more portable ways (Francesko et al., 2018; Weller et al., 2020).
